# Honor classes in China's higher education: perspectives from four universities

**DOI:** 10.1093/nsr/nwac002

**Published:** 2022-01-07

**Authors:** Weijie Zhao

**Affiliations:** NSR, news editor based, Beijing

## Abstract

Ever since the University of Science and Technology of China started the Special Class for the Gifted Young in 1978, many Chinese universities have set up their own honor classes under different names such as Gifted Young Class, Basic Science Class, Experimental Class or Elite Class. These are often small classes; some emphasize mathematics and physics, aiming to cultivate talents with broad basic knowledge, while others focus on a particular discipline, such as computer science. In 2011, the Chinese Ministry of Education started the Experimental Program for the Cultivation of Top Talents in Basic Sciences, which boosted a new round of honor classes.

How do these classes cultivate their students? Which kinds of special strategies are employed? Have they indeed fostered outstanding graduates? How did the students develop after graduation? On the other hand, are these classes a violation of the spirit of ‘equity in education’? Trying to answer these questions, in this *NSR* forum chaired by Professor Zhenjiang Hu, educators from four Chinese universities exchanged their experiences, views and perspectives on running the honor classes.

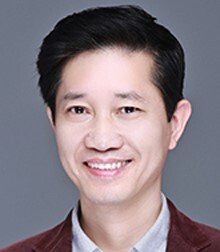

Baoquan Chen

Executive Director of the Center on Frontiers of Computing Studies, School of Electronics Engineering and Computer Science, Peking University. (Turing Class, Peking University.)

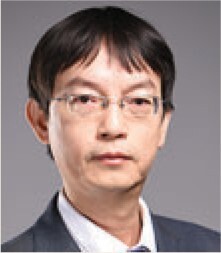

Changqing Chen

Professor and Head of the Department of Engineering Mechanics, Tsinghua University. (Tsien Excellence in Engineering Program, Tsinghua University.)

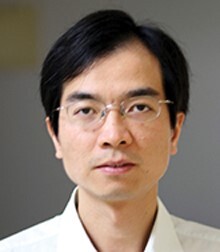

Zhenyu Li

Professor of the School of Chemistry and Materials Science, University of Science and Technology of China. (School of the Gifted Young, University of Science and Technology of China.)

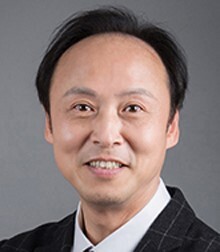

Yong Yu

Professor of the Department of Computer Science and Engineering, Shanghai Jiao Tong University. (ACM Class, Shanghai Jiao Tong University.)

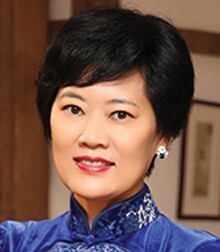

Ming Zhang

Chair of the ACM Special Interest Group of Computer Science Education (SIGCSE) China; Professor of the School of Electronics Engineering and Computer Science, Peking University.

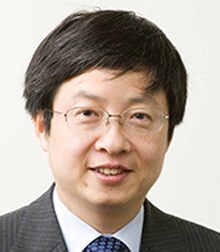

Zhenjiang Hu (Chair)

Dean of School of Computer Science, Peking University.

## THE BEGINNING OF THE HONOR CLASSES

**ZJ Hu**: Thank you all for joining this panel discussion! Firstly, please introduce the honor class in which you are engaged. How was it founded? What are its original goals?

**BQ Chen:** The Turing Class of Peking University (PKU) is relatively young. It was founded in 2017, after the 1986 Turing Prize winner John Hopcroft became a Visiting Chair Professor of PKU. We enrolled 30 students from Grade 2016 for the first Turing Class, and now the students are basically enrolled from the freshmen, including high-school-contest prize winners and top scorers in the National College Entrance Examination. There are also a few from the sophomore class.

John has many ideas for undergraduate computer education, but it's difficult to try these new ideas in an entire School of Computer Science because undergraduate education is extremely important and complex. John's original intention for the Turing Class was not to offer ‘elite education’ to a small group, but to test his educational ideas. He explained several times that he wished to extend the successful approaches

The Tsien Excellence in Engineering Program (TEEP) of Tsinghua University (THU) was co-designed by eight engineering departments at THU.

—Changqing Chen

of the Turing Class to the education of all computer science students.

**M Zhang:** I would like to add that before the Turing Class, PKU’s School of Electronics Engineering and Computer Science already had some honor classes.

PKU started to enroll high-school-computer-contest prize winners around 2003, and we immediately noticed that there was a big gap between the general students and ‘the prize winners’ in terms of basic knowledge and computer skills. In 2003, most Chinese families did not have a computer and many of the freshmen had seldom touched a keyboard, while it was apparent that this was not the case for the prize winners. When we gave the same courses to them, the prize winners felt bored, while many other students experienced pressure from these peers.

In 2006, the school arranged for several teachers to visit Hong Kong. We noticed that there were honor classes in Hong Kong universities, so we started experimental classes in 2007. The first two were the special courses ‘Introduction to Computing’ and ‘Data Structures and Algorithms’, each consisting of about 20 students. After that, we set up several more experimental classes for several core courses.

In 2011, the Chinese Ministry of Education started the Experimental Program for the Cultivation of Top Talents in Basic Sciences. With the funding of this program, we set up the Top-Tier Class. Based upon the original series of experimental classes, we added scientific research guidance for the top talents and were able to provide better resources for them, for example, we began organizing visits to the US universities and funding the students for international conferences so that they could have an international vision and touch the research frontiers at an earlier stage.

**Y Yu:** I have been teaching at Shanghai Jiao Tong University (SJTU) since 1986. Contacting the students, I noticed a number of problems. The Association for Computing Machinery (ACM) International Collegiate Programming Contest (ICPC) came to China in 1996, and SJTU was among the first Chinese universities taking part in this contest. I noticed even more problems during the contest and began to consider the possibility of taking advantage of the ACM contest to create a special class, in which I could cultivate the students using some of my ideas. In 2002, SJTU won the ICPC World Championship. I took the opportunity to file my proposal with the university and the ACM Class started.

The ACM Class was founded in the name of ICPC, but it is definitely not a class for contest. Our aim is to foster computer scientists. Many Chinese students have got medals in all kinds of international contests, but very few of them have become outstanding scientists. I hope to make some attempts and do some exploration. In 2011, John Hopcroft joined SJTU, which made the ACM Class much more international.

**CQ Chen:** The Tsien Excellence in Engineering Program (TEEP) of Tsinghua University (THU) was co-designed by eight engineering departments at THU, and officially started in 2009. At that time, THU already had honor classes for basic mathematics and physics, as well as the ‘Yao Class’ for computer science, but an honor class for engineering was missing. Mechanics is considered to be the basis of all engineering disciplines, so the eight departments agreed to create TEEP under the School of Aerospace Engineering as the honor class for engineering.

The major goal of TEEP is to cultivate innovative research talents. It is relatively easy for the students to learn facts, but difficult for them to think outside the box and create new knowledge. We hope that by applying special courses and training strategies we can arouse the initiative of the students and guide them with regard to innovative research works.

**ZY Li**: The Special Class for the Gifted Young (SCGY) of the University of Science and Technology of China (USTC) is somewhat different from the above-mentioned classes, for SCGY has a relatively long history and is not a class for certain disciplines. SCGY was founded in 1978, when our nation lacked proper education vehicles for young talents. At that time, a high school teacher introduced a 13-year-old ‘child prodigy’ to the Chinese Academy of Sciences (CAS), so with the promotion of CAS and the Ministry of Education, SCGY was set up in USTC to offer specific education to these gifted students younger than most undergraduates.

Throughout the decades, the major philosophy of SCGY has been ‘to teach students in accordance with their aptitude’. There are so many students in China, and for a tiny gifted fraction of them, finishing three years of high school education and then going to college at 18 years old is not the best choice. If they can enter college at an earlier age, they may have a better future. SCGY is offering such opportunities, and a personalized education, to them.

Today, SCGY is not a single class—we established the School of Gifted Young (SGY) in 2008. There are three types of classes in SGY: the traditional SCGY (freshmen no more than 16 years old), the Innovation Pilot Class (freshmen no more than 17 years old), and the Experimental Class for Basic Science (selected from the general freshmen). SGY plays two roles in USTC: a school to foster top talents and a test bed for educational reform.

SCGY was founded in 1978, when our nation lacked proper education vehicles for young talents.

—Zhenyu Li

Besides SGY, USTC has several other honor classes for certain majors, which are co-managed with CAS institutes. For example, the Mathematics Honor Class is supported by the CAS Academy of Mathematics and Systems Science, and the Physics Honor Class is supported by the CAS Institute of Physics. These classes were originally placed under SGY, but we soon realized that it would be more appropriate to run them under corresponding departments.

## STRATEGIES AND OUTCOMES

**ZJ Hu**: Thank you. Now please introduce your educational strategies and outcomes. How do you cultivate gifted students, and have they become outstanding graduates?

**ZY Li:** SGY students are not assigned to any department in the freshman year. In this year, they have hardcore courses to lay a solid foundation of mathematics and physics, and are introduced to different majors so that they can make rational choices at the end of the year. We also rearranged the textbooks to fit with this special strategy.

SGY has a motto of ‘Freedom, Independence and Confidence’. We encourage the students to independently explore their interests, and help them build up confidence. The confidence they have due to outstanding academic performance in high school may be fragile. Only when they have actually explored their own interests and strengths are they able to construct real confidence that can benefit them for a lifetime.

SGY has advantages in cultivating interdisciplinary talents. Because a student's classmates and roommates choose different majors, he or she has a circle of friends in all these disciplines. As a result, it is easier for the student to switch their major or perform interdisciplinary research. Indeed, we have a special class for interdisciplinary talents in SGY. Students in this class can select courses from several different departments according to their own interests, and they can choose to finish their course in four or five years.

Regarding the outcome, SGY has fostered many high-quality talents. In the world of academia, 5 out of the first batch of 1000 SGY graduates became academicians. This ratio is much higher than USTC as a whole. Many famous scientists are SGY alumni, such as Professor Xiaowei Zhuang of Harvard University, who is legendary for earning full marks in all of the four major physics courses. In the younger generation, we also have fostered outstanding researchers such as Professor Yunji Chen of CAS University and Dr. Yuan Cao, currently a postdoc at Harvard. As a graduate student at MIT, Cao has made world acclaimed scientific breakthroughs in the study of graphene. In the industrial world, our graduates include Biao Wan, CEO of HONOR, and Tianshi Chen, founder and CEO of Cambricon Technologies.

**BQ Chen:** The Turing Class is for people majoring in computer science, so our strategies follow the requirements of this scientific discipline: firstly, we emphasize the importance of mathematics and physics; secondly, computer science is a practical science so the students need to practice as much as possible.

To offer practical training, we set up a whole-year course of research practice for second-year students. In this course, we not only introduce the research directions of computer science and teach the basic principles of research methods and research ethics, but most importantly, the students are required to do a research rotation in three different labs. They can select the rotation labs within our mentor pool, which consists of around 60 mentors from in and out of PKU. They spend six weeks in each lab to experience real research, and after that, at the end of the second year, they choose a lab in which to stay for further research. Importantly, we request that the mentors leave the selection to the students. A mentor should not consider a student to be his or her own during the rotation.

In summer, after the third year, Turing Class students have a chance to go abroad. They contact foreign professors with the help of their native mentors, and work in the foreign lab for a summer, or for half a year.

We have a committee for the management of the Turing Class, and the John Hopcroft Foundation to accept and manage social donations. The foundation is more flexible compared to the national programs, and it plays a complementary role in funding the students’ research activities.

Started in 2017, the Turing Class has only two years of graduates. Among the 30 students of the first Turing Class, almost everyone published a research article, as the first author, during the four years. And after graduation, almost everybody continued to pursue a PhD or master's degree, some in PKU, and most in reputable Western universities. So we can say that the Turing Class is performing well in the cultivation of future scientists.

The aim of the ACM Class is to cultivate computer scientists, so we emphasize the importance of mathematics and practice.

—Yong Yu

**M Zhang:** Here is a little data from the PKU Top-Tier class before the Turing Class. Before 2007, of all the Computing graduates from PKU’s School of Electronics Engineering and Computer Science, less than 10 students would go on to earn a PhD degree each year, and only one or two would become scientists and find jobs in universities. But now, most graduates go to graduate schools, and among the 2007 students, six have already got faculty-track positions in universities in and out of China.

**Y Yu**: The aim of the ACM Class is to cultivate computer scientists, so we emphasize the importance of mathematics and practice. Our students usually enter laboratories in their second year. Some students would like to go into a lab in the first year, but I do not agree with that. Because for the freshmen who are good at coding but have little sense of real computer science research, if they enter labs in the first year, some mentors would use them as mere coders, which would damage their long-term development.

Our students also go abroad for research internships in the summer of the third year. In the earlier years, John helped to contact Cornell University for summer visits, but now, students apply by themselves with some help from the teachers. It's good training for their future research careers.

One problem is that many students are confused as freshmen. They do not know much about their major, and do not understand the relationship between the basic courses and their future research, so many of them waste one or two years before they figure out their path. To address this issue, I spent a lot of effort on ideological and mental education, hoping the students become interested in their major as soon as possible and start to learn and research actively and independently.

Furthermore, we have a course called ‘Student's Lecture’. For as long as two years, every student should give a lecture once a semester and the topic should be unrelated to their major. Through this course, I wish to improve their self-confidence and cultivate their ability to communicate, which is a significant academic ability that is often ignored by Chinese students.

After graduating, more than 95% of ACM Class graduates pursue a master’s or PhD degree, mostly in the top Western universities. I strongly recommend they go abroad, because I believe that a modernized, competitive talent should understand not only China, but also the mainstream scientific culture of the world.

By now, there have been at least 25 ACM Class graduates that have become faculty members in various universities in and out of China. In the industrial world, we have fostered the senior chief scientist of Amazon and the AI Lab leader of ByteDance, as well as the co-founders of 4Paradigm, LAIX and CooTek—LAIX and CooTek have gone public on the New York Stock Exchange.

After about 20 years of practice, the ACM Class has entered a stage of sound development. Many graduates have begun to support the Class, in turn, with their resources.

**CQ Chen:** TEEP also applies similar practices to motivate students’ initiative with regard to learning and researching. THU has a traditional Student Research Training (SRT) program, in which undergraduates can choose a mentor and perform a small research project. But in the early years, this program was not treated seriously by either the students or the teachers. In TEEP, however, each student has to do one or two SRT programs conscientiously in the first two years, and after that, they can propose and perform their own research plans under the Open Research for Innovative Challenges (ORIC) program. Similar to the Turing Class, we have a mentor team consisting of professors from not only the eight engineering departments, but also other THU departments and even other universities. After this research training, we also offer the students opportunities to study abroad.

We set up several courses to improve students’ comprehensive quality, one of which invites experts from diverse fields such as music and Brain Projects to broaden the vision of our students.

During the four years in TEEP, about half the students publish a research article, many in renowned journals. After graduation, about one third go abroad for further study, and the other two thirds pursue higher degrees in different departments of THU.

TEEP started in 2009 and the first students have obtained their PhD degrees for about two years. Within such a short time, two of them have already become professors. Now, TEEP is not only a special class for mechanics, but also a basic class for a wide range of engineering subjects.

But at the same time, we noticed some problems. The students enrolled in TEEP are all excellent high-school graduates. But every year there are one or two students who lose motivation and cannot catch up with the schedule. In the second or third year, these students are reassigned to general classes in different departments. After the reassignment, some perform better or even excellently, but some remain dispirited. How to solve this problem? I think we need more deliberation.

The US honor classes have several main strategies: first, the mentor system … ; second, the small-class strategy; third, personalized education …

—Ming Zhang

**ZJ Hu**: Thanks for offering so many details. Professor Zhang, would you please say a little more about the honor classes in Western countries? What can we learn from them?

**M Zhang:** Honor education has a long history in Western countries. Honor classes exist at all levels of education—from primary schools to universities. They appeared first in Europe and then in the US. Honor classes are a prominent mark of US higher education. The classes have fostered a large number of outstanding scientists, entrepreneurs and politicians. For example, most winners of the Sloan Research Fellowship and other prizes for young researchers are honor class graduates.

The US honor classes have several main strategies: first, the mentor system—every student has his or her own mentor; second, the small-class strategy; third, personalized education—they schedule personalized curricula and research plans for each student. The Chinese special classes have tried to adopt all these strategies, and additionally, we need to emphasize internationalization, to provide our students with an international vision and opportunities to make contact with the world’s research frontiers.

Recently, I participated in writing Computing Curricula 2020, which is produced by ACM and IEEE computing education experts every 15 years, and is an important guide for global computing education. The previous version is well known as ACM/IEEE CC2005. The report does not particularly emphasize honor education, but it proposes a ‘Competency Model’, where the ‘competency’ consists of three aspects: knowledge, skill and disposition. The ‘disposition’ here is similar to the ‘ideological and mental education’ mentioned by Professor Yu, which forms the basis for the students’ lifelong sustainable learning.

## ARE THESE CLASSES AFFECTING EDUCATION EQUITY?

**ZJ Hu**: What's the relationship between the honor classes and the general classes? Are the honor classes running against the ideal of equity in education?

**CQ Chen:** The honor classes are sometimes considered to be ‘elite classes’ in China, but I think this is not appropriate, because it's complicated to define ‘elite’, and to foster ‘elites’ is not the only aim of these classes. Actually, as we have mentioned above, these classes are experimental classes and the educational strategies may be applied to general classes.

In the sense of resource allocation, it is true that in the earlier years, TEEP enjoyed more educational resources than the general classes. But now, more special educational programs are emerging in Chinese universities, so that more students are enjoying resources similar to that of TEEP. I believe that in the next one or two decades, China's higher education will undergo tremendous changes, and many of the new strategies will originate from the experiences of the honor classes.

**M Zhang:** I agree that the educational strategies of the honor classes can be extended to general classes. In our school, we have extended the mentor system to all students by assigning a mentor to everyone.

On the other hand, I want to point out that the College Entrance Examination itself is a selective examination, whose aim is to sort high-school graduates into different universities and different majors that are suitable for them. Of course, if we have tens or hundreds of high-level universities—just like the US—we will be able to better allocate our educational resources and offer high-level personalized education to more students.

**ZY Li:** I want to comment on two aspects. Firstly, I think ultimate fairness in education means to educate students in accordance with their distinct aptitudes. If we teach exactly the same courses at the same pace to everyone, a group of students may feel it is too easy, while another group may have difficulty catching up. Such a uniform arrangement actually would not be fair to both groups.

Secondly, we cannot say that the honor classes seize resources from the general classes. Instead, the special education plans developed for the gifted classes can be a valuable resource enjoyed by the general student population as well. For example, in 2020 we started to record and post online at Bilibili [a video website in China] actual classes of the SGY students, taught by the most talented lecturers at USTC. So far, four courses in calculus and introductory physics have been posted, and they proved to be popular among college and high school students nationwide.

I think ‘honor class’ is an appropriate name, because the graduates of these classes do have a sense of honor, which provides them with higher self-expectation so that they are more likely to realize their full potentials.

USTC is also extending the practices of SGY to the general classes with some adaptations. For example, the general students are also given a chance to switch major at the end of the freshman year.

**Y Yu**: Students are different, so it is fair to educate them differently. If we refuse to foster the gifted students into real talents in the name of ‘equity’ it would be a tragedy.

Regarding the relationship between different classes, the honor classes can naturally influence the general ones, because the general class students who want to improve themselves would observe, learn and imitate the practices of the honor class students. For example, since the foundation of the ACM Class there have been more general class students in our department who go abroad for further education, which is a result of their own effort and choice.

**BQ Chen:** I think education equality may be a pseudo proposition. Because good higher education should be personalized, and personalized education is unlikely to be identical for every student. For example, if a student is interested in building quantum computers, and we want to offer him or her personalized education, then the resources we spend on this student would definitely be different from the other students.

I agree that the special classes are a kind of exploration, and more importantly, they are an exploration of how to do personalized education. Actually, every student needs personalized education, and our current issue is how to extend personalized educational practices from the honor classes to more students. Professor CQ Chen mentioned that some students in TEEP cannot catch up with their classmates. There are also similar situations in the Turing Class. We should think more about how to offer these students an educational environment that is more suitable for them.

Our current issue is how to extend personalized educational practices from the honor classes to more students.

—Baoquan Chen

## THE NEXT STEPS

**ZJ Hu**: Thank you! Our last question is: what should be the next steps of these special classes?

**BQ Chen:** I think in the future our honor classes should be more flexible. Currently, these gifted classes offer high-quality resources in all aspects, from courses and research opportunities to international perspectives. But in the future, especially when we try to extend personalized education to more students, we can offer these high-quality resources to different students according to their own interests and characteristics. For example, we can offer some students advanced courses and others research opportunities. I think the flexible mode of education will become a trend.

Furthermore, from my own observation, the most important educational resource is the teachers. Without outstanding lecturers and mentors it is impossible to foster outstanding students. From my perspective, an outstanding teacher is not just a good scientist, but more importantly, he or she should have advanced educational ideas and be willing to devote themselves to education. We need a group of such teachers to keep on practicing and communicating with each other, to come up with new ideas and improve our higher education.

**CQ Chen:** My greatest expectation is that we use the experiences of the honor classes to benefit more students. The four universities involved in today's forum are all top Chinese universities. I believe that all students in these universities can be considered as elite and deserve a high-level personalized education.

Now, we are preparing to construct a ‘Zero-One College’ in Shenzhen, whose aim is to apply the experiences of TEEP to the education of more students. China needs a large number of talents and that requirement cannot be fulfilled by the current small-sized honor classes.

**ZY Li:** I noticed that the mode of SGY is different from the mode of your classes. Broad-discipline education and discipline-focused education each have their own strengths. So maybe we should think about how to combine these strengths to foster even better talents covering both aspects, a broad base of scientific knowledge and the ability to dig deep into a specific discipline.

**Y Yu:** I think a question is, whom should the talents we foster service? How can we educate our gifted students so that they will be willing to pay back to the nation and society that have fostered them? We should think more about these issues. I also agree with the importance of teachers. And finally, I think communication, like this panel discussion today, is very useful, we can learn from each other and come up with new ideas.

**M Zhang:** To sustainably develop in the future, honor education in China should better integrate all kinds of resources, including national policies, teachers and the alumni resources in and out of China. Resources of different universities should also integrate, so that we can build a larger and healthier talent-cultivating environmental system out of the whole nation.

**ZJ Hu**: Thank you very much for your discussion, for the experiences you shared and for your opinions on educational fairness and the future direction of China's honor education system. I believe these ideas will be a good reference point for many higher educators in China.

